# Preoperative administration of intramuscular dezocine reduces postoperative pain for laparoscopic cholecystectomy

**DOI:** 10.1016/S1674-8301(11)60047-X

**Published:** 2011-09

**Authors:** Yaomin Zhu, Guixia Jing, Wei Yuan

**Affiliations:** Department of Anesthesiology, the First Affiliated Hospital of Medical College, Xi'an Jiaotong University, Xi'an, Shaanxi 710061, China

**Keywords:** dezocine, postoperative pain, laparoscopic cholecystectomy

## Abstract

Postoperative pain is the most common complaint after laparoscopic cholecystectomy. This study was carried out to evaluate whether preoperative administration of intramuscular dezocine can provide postoperative analgesia and reduce postoperative opioid consumption in patients undergoing laparoscopic cholecystectomy. Patients (ASA I or II) scheduled for laparoscopic cholecystectomy were randomly assigned into intramuscular dezocine group (group 1) or intramuscular normal saline group (group 2). Dezocine and equal volume normal saline were administered intramuscularly 10 min before the induction of anesthesia. After operation, the severity of postoperative pain, postoperative fentanyl requirement, incidence and severity of side-effects were assessed. Postoperative pain and postoperative patient-controlled fentanyl consumption were reduced significantly in group 1 compared with group 2. The incidence and severity of side effects were similar between the two groups. Preoperative single-dose administration of intramuscular dezocine 0.1 mg/kg was effective in reducing postoperative pain and postoperative patient-controlled fentanyl requirement in patients undergoing laparoscopic cholecystectomy.

## INTRODUCTION

Postoperative pain is the most common complaint[1],[2] and the primary reason for prolonged convalescence[3],[4] after laparoscopic cholecystectomy. Intense acute pain after laparoscopic cholecystectomy could predict the development of chronic pain[5],[6]. Dezocine, a synthetic bridged aminotetralin with minimum side effects and low dependence liability, is a parenterally administered opioid analgesic that has both agonistic and antagonistic actions at opioid receptors[7],[8]. Dezocine was found to be 7 to 18 times as potent as morphine, and demonstrated slightly less antagonistic activity than nalorphine[9],[10]. One study showed that dezocine did not suppress abstinence in withdrawn morphine-dependent monkeys, nor did it produce dependence when administered chronically in monkeys[10]. Some studies showed that dezocine was found to possess less potential for producing bronchoconstriction, respiratory depression, hypotension, and histamine-release than either morphine or pentazocine[11],[12]. In humans, dezocine was 8.6 times as potent as pentazocine in terms of respiratory depressant effects[13]. In two clinical studies on the drug's analgesic properties, the potency of 10 mg of dezocine was considered to be at least that of 50 mg of meperidine and of 10 mg of morphine[14],[15]. However, there are no reports on whether preoperative dezocine can reduce postoperative pain and the postoperative opioid requirement after laparoscopic cholecystectomy surgery.

This study was carried out to evaluate whether preoperative administration of intramuscular dezocine can provide postoperative analgesia and reduce postoperative opioid consumption in patients undergoing laparoscopic cholecystectomy under general anesthesia.

## MATERIAL AND METHODS

### Subjects

The study protocol was approved by the Institutional Human Ethics Committee of the First Affiliated Hospital of Medical College, Xi'an Jiaotong University, Xi'an, Shaanxi, China. Written informed consent was obtained from each patient. Based on a random, double blind design and control methods, 60 patients (ASA physical status I or II) scheduled for laparoscopic cholecystectomy were enrolled in this study. The patients were between 33 and 65 years old. The subjects were randomly assigned into two groups of 30 each with the help of a computer-generated table of random numbers, to receive either intramuscular dezocine 0.1 mg/kg (group 1) or normal saline in equal volume (group 2). Patients with history of chronic pain or daily intake of analgesics, uncontrolled medical disease (diabetes mellitus and hypertension), and inability to operate patient-controlled analgesia (PCA) device were excluded from the study. All the medications were provided by hospital pharmacy, and were identical.

### Anesthesia

Dezocine and equal volume of normal saline were administered intramuscularly, 10 min before the induction of anesthesia by a staff nurse who was not involved in the study. Anesthesia technique was standardized in all the groups. Patients received totally intravenous anesthesia and mechanical ventilation. Anesthesia was induced with 4 µg/mL propofol by target-controlled-infusion (TCI), 3 µg/kg fentanyl, and 0.05 mg/kg midazolam. Neuromuscular blockade was achieved with 0.1 mg/kg vecuronium and the trachea was intubated. Anesthesia was maintained with propofol by TCI, remifentanil was continuously infused at the rate of 0.5-1.5 µg/(kg·min). Percutaneous oxygen saturation was maintained at 98% or more, and end-tidal carbon dioxide tension was maintained at 35 mmHg during surgery. The depth of anesthesia was maintained with the bispectral index at a score of 40-50 to ensure similar anesthetic depth in all patients. Acetated Ringer's solution was infused at a rate of 6 to 8 mL/(kg·h) during surgery. In group 2, one patient who underwent conversion to open cholecystectomy was considered as drop-out and was therefore not included for further study. After satisfactory recovery, the patients were extubated and received intravenous injection fentanyl *via* PCA pump with an activated dose of 20 µg with a lockout interval of 15 min.

### Clinical evaluation

The severity of postoperative pain, postoperative fentanyl requirement, incidence and severity of side-effects such as postoperative nausea and vomiting (PONV), headache, sedation, and respiratory depression were all assessed by an independent anesthesia registrar blinded to group allocation.

Assessment of pain both at rest and during coughing was done by a 100 mm visual analogue scale (VAS): 0, no pain; 100, worst imaginable pain[16]. VAS was measured at 0, 4, 8, 12, and 24 h after surgery by a trained nurse blinded to the study drug. The severity of PONV[17] was graded on a four-point ordinal scale (0, no nausea or vomiting; 1, mild nausea; 2, moderate nausea; 3, severe nausea with vomiting). Rescue antiemetic ondansetron 4 mg intravenous injection was given to all patients with PONV of grade ≥ 2. The Ramsay sedation scale (1, anxious, agitated, or restless; 2, cooperative, oriented, and tranquil; 3, response to command; 4, brisk response; 5, a sluggish response; 6, no response) was used to assess sedation; patients with a sedation scale of ≥4 were considered as sedated[18]. Respiratory depression was defined as ventilatory frequency ≤ 8 bpm and oxygen saturation < 90% without oxygen supplementation[19].

### Statistical analysis

The subject who underwent conversion to open cholecystectomy in group 2 was not subjected to further statistical analysis. The SPSS 15.0 program was used to analyze the statistical data. Values are expressed as mean±SD, or the number of patients. Patient characteristic data were analyzed with one-way ANOVA for continuous variables and Chi-square test for categorical variables. The VAS pain scores and postoperative PCA fentanyl consumption were analyzed with Student's *t*-test. The incidences of side-effects and sedation were analyzed with Fisher's exact test. Signiﬁcance was determined at *P* < 0.05.

## RESULTS

### Baseline conditions

One subject who underwent conversion to open cholecystectomy was considered as drop-out after initial randomization and was therefore not included for further statistical analysis. There were no significant differences between the two groups with respect to age, body weight, sex, duration of anesthesia and duration of surgery (***Table 1***).

**Table 1:** Patient general characteristics

	Group 1 (*n*=30)	Group 2 (*n*=29)
Age (y)	44 ± 9	43 ± 10
Weight (kg)	64±12	63 ± 10
Sex (M/F)	18/12	16/13
Duration of anaesthesia (min)	62 ± 31	65 ± 33
Duration of surgery (min)	38 ± 25	40 ± 24

Group 1: patients who received intramuscular dezocine. Group 2: patients who received intramuscular saline at an equal volume as dezocine. There were no significant differences between group 1 and 2.

### VAS scores and fentanyl consumption after surgery

**Fig. 1:**
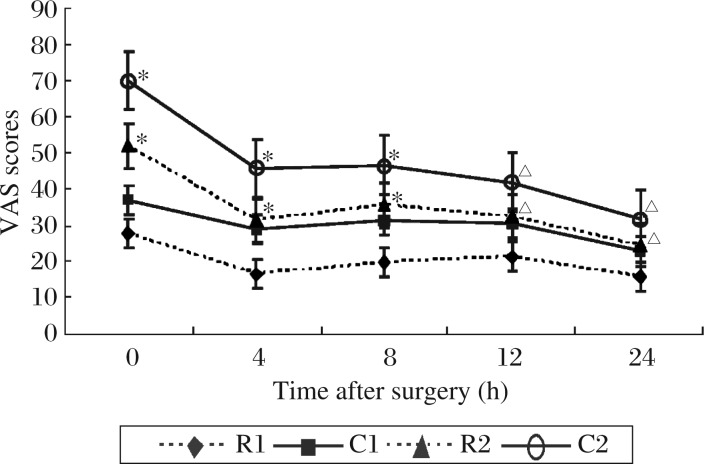
Postoperative VAS scores at 0, 4, 8, 12, and 24 h after surgery in two groups. R2 compared with R1, C2 compared with C1, ^△^*P* < 0.05 and *P < 0.01. VAS: visual analogue scale (0, no pain; 100, worst imaginable pain). R1: VAS scores at rest in group 1. C1: VAS scores during coughing in group 1. R2: VAS scores at rest in group 2. C2: VAS scores during coughing in group 2. Group 1: patients who received intramuscular dezocine. Group 2: patients who received intramuscular saline at an equal volume as dezocine.

The VAS scores at 0, 4, 8, 12, and 24 h after surgery at rest in group 1 were significantly lower than those in group 2; the VAS scores at 0, 4, 8, 12, and 24 h after surgery during coughing in group 1 were significantly lower than those in group 2 (***Fig. 1***). Postoperative patient-controlled fentanyl consumption were reduced significantly in group 1 (458.5±89.7 µg) compared with group 2 (856.3±101.2 µg, *P* < 0.01).

### Sedation scores

**Fig. 2:**
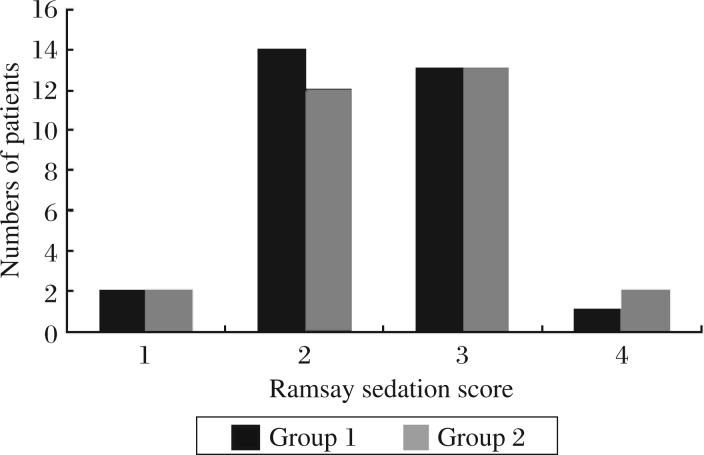
The distribution of patients in the different scales of Ramsay sedation scores of the two groups. There were no significant differences between group 1 and 2. Ramsay sedation score (1: anxious, agitated, or restless; 2: cooperative, oriented, and tranquil; 3: response to command; 4: brisk response). Group 1: patients who received intramuscular dezocine. Group 2: patients who received intramuscular saline at an equal volume as dezocine.

There were no significant differences in the number of patients in the different scales of Ramsay sedation scores (1, 2, 3 and 4) between two groups (***Fig. 2***).

### The incidence of side-effects

**Fig. 3:**
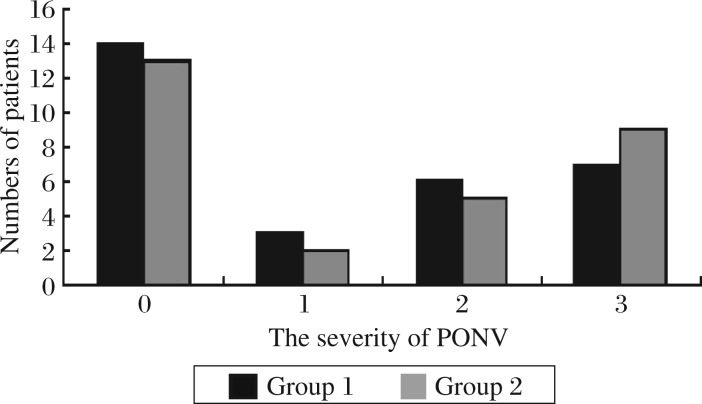
The distribution of patients in the different grades of PONV of the two groups. There were no significant differences between group 1 and 2. PONV: postoperative nausea and vomiting; 0: no nausea or vomiting; 1: mild nausea; 2: moderate nausea; 3: severe nausea with vomiting. Group 1: patients who received intramuscular dezocine. Group 2: patients who received intramuscular saline at an equal volume as dezocine.

There were no significant differences in the number of patients in the incidence and severity of PONV (0, 1, 2 and 3) between the two groups (***Fig. 3***). The number of patients requiring antiemetics, incidence of headache, and respiratory depression were similar between two groups.

## DISCUSSION

Dezocine is an analgesic agent with opioid agonist and antagonist activity[20]. After parenteral administration of therapeutic doses, it is approximately equipotent with morphine, and has proved at least as an effective analgesic as morphine, pethidine and butorphanol in moderate to severe postoperative pain[21]. However, preliminary pharmacodynamic data indicate that the ceiling of analgesic activity of dezocine occurs at a higher level of analgesia than that of reference agonist/antagonist agents. Additionally, the drug exhibited a morphine-like degree of anaesthetic-sparing activity in animals. Although long term data are very limited, single doses of dezocine are well tolerated, with mild and transient sedation and gastrointestinal upset the principal adverse effects[22]. In single analgesic doses, dezocine is a slightly more potent respiratory depressant than morphine[23]. Clinically, important haemodynamic changes have not been observed with usual analgesic doses of dezocine. As an agonist/antagonist opioid, the dependence liability of dezocine would be expected to be lower than that of pure agonist opioids[24].

In our study, we observed that the VAS scores of postoperative pain at 0, 4, 8, 12, and 24 h after surgery and the doses of postoperative patient-controlled fentanyl consumption after surgery were reduced significantly in group 1 when compared with group 2. These results indicate that preoperative single-dose administration of intramuscular dezocine was effective in reducing both the static and the dynamic components of postoperative pain along with postoperative patient-controlled fentanyl consumption in subjects undergoing laparoscopic cholecystectomy. Camu *et al*.[25] showed that dezocine 10 mg was less effective than meperidine but dezocine 15 mg showed a rapid onset of analgesic effect with long-lasting analgesia superior to meperidine. In Cohen *et al*.'s study[26], adult patients who had arthroscopic surgery under general anesthesia and requested postoperative pain relief were randomized to receive treatment in a double-blind protocol with 5 mg of intravenous dezocine, morphine, nalbuphine, or saline. The results showed that dezocine and morphine are more efficacious than nalbuphine in the management of early postoperative pain. As an alternate analgesic in this study, dezocine required fewer doses to achieve patient satisfaction and was thus more efficacious than morphine. In Ding *et al*.'s study[27], patients undergoing outpatient laparoscopic procedures received ketorolac (60 mg) or dezocine (6 mg) or fentanyl (100 µg) before the start of the operation. In the postanesthesia care unit, 61% of patients in the fentanyl group received analgesic drugs for persistent pain, compared with 34% and 25% in the ketorolac and dezocine groups, respectively, and less postoperative fentanyl was required in the ketorolac (22±33 µg) and dezocine (18±35 µg) groups, compared with the fentanyl (58±33 µg) group. The study by Finucane *et al*.[28] indicates that a single 10 or 15 mg intramuscular injection of dezocine is safe and more effective than placebo for 4-6 h, respectively, in the treatment of moderate to severe postoperative pain, and both doses of dezocine provided long-lasting relief. The scores on all three efficacy scales were the highest with the 15 mg dose of dezocine after the first hour.

Our study showed that, in group 1, dezocine was administered intramuscularly 10 min before the induction of anesthesia; in group 2, equal volume saline was administered intramuscularly 10 min before the induction of anesthesia. The results showed that the severity of postoperative pain was reduced significantly at 0, 4, 8, 12, and 24 h after operation and postoperative fentanyl requirement was also reduced significantly in group 1 compared with group 2. The reason may be that dezocine administered intramuscularly before operation can produce pre-emptive analgesia and reduce the fentanyl doses during postoperative PCA in patients undergoing laparoscopic cholecystectomy. Dah *et al*. [29] showed that pre-emptive analgesia has the potential to be more effective than a similar analgesic treatment initiated after surgery, and the immediate postoperative pain may be reduced and the development of chronic pain may be prevented.

Our data showed that there were no significant differences in the incidence and severity of sedation between the dezocine group and control group. The reason may be that the dose of dezocine which we used or the dose of fentanyl by patient-controlled consumption is small. Ramirez-Ruiz *et al*.[30] showed that compared with ketorolac 60 mg, fentanyl 100 µg and dezocine 6 mg produced a greater decrease in the propofol sedation requirement during monitored anesthesia care. In Zacny *et al*.'s study[31], 10 healthy volunteers (six men and four women) were injected with 0, 2.5, 5.0, and 10 mg of dezocine in a double-blind fashion, and their results indicate that dezocine had a sedative effect in a dose-dependent fashion.

In our study, the incidence and severity of PONV, number of patients requiring antiemetics, incidence of headache, and respiratory depression were similar between the two groups. Camu *et al*.[25] found that vital signs remained stable within satisfactory limits with no respiratory depression occurring after administration of 10 or 15 mg dezocine, side effects observed appeared to be dose-related. Some researchers found that dezocine was associated with an increased incidence of postoperative nausea and a delayed discharge time compared with ketorolac, and with an decreased incidence of postoperative nausea compared with morphine[27],[32]. The difference in their results from our study could possibly be because we administered a smaller dose of dezocine against their starting dose or because of the reduced postoperative patient-controlled fentanyl consumption or the difference in the nature of surgery. Limitations of the present study are that we did not evaluate the dose–response or the effect of continuation of therapy. Further studies are suggested in these areas.

In conclusion, preoperative single-dose administration of intramuscular dezocine 0.1 mg/kg was effective in reducing postoperative pain and postoperative patient-controlled fentanyl requirement in patients undergoing laparoscopic cholecystectomy. The side-effect proﬁle was similar in both groups. We therefore suggest that preoperative single-dose administration of intramuscular dezocine is an effective method for reducing postoperative pain and fentanyl consumption in patients undergoing laparoscopic cholecystectomy.
